# Supplementary figures and images for: Integrating comprehensive functional annotations to boost power and accuracy in gene-based association analysis
Source: PLoS Genet. 2020 Dec 15;16(12):e1009060. doi: 10.1371/journal.pgen.1009060 (PMC7737906; doi:10.1371/journal.pgen.1009060)

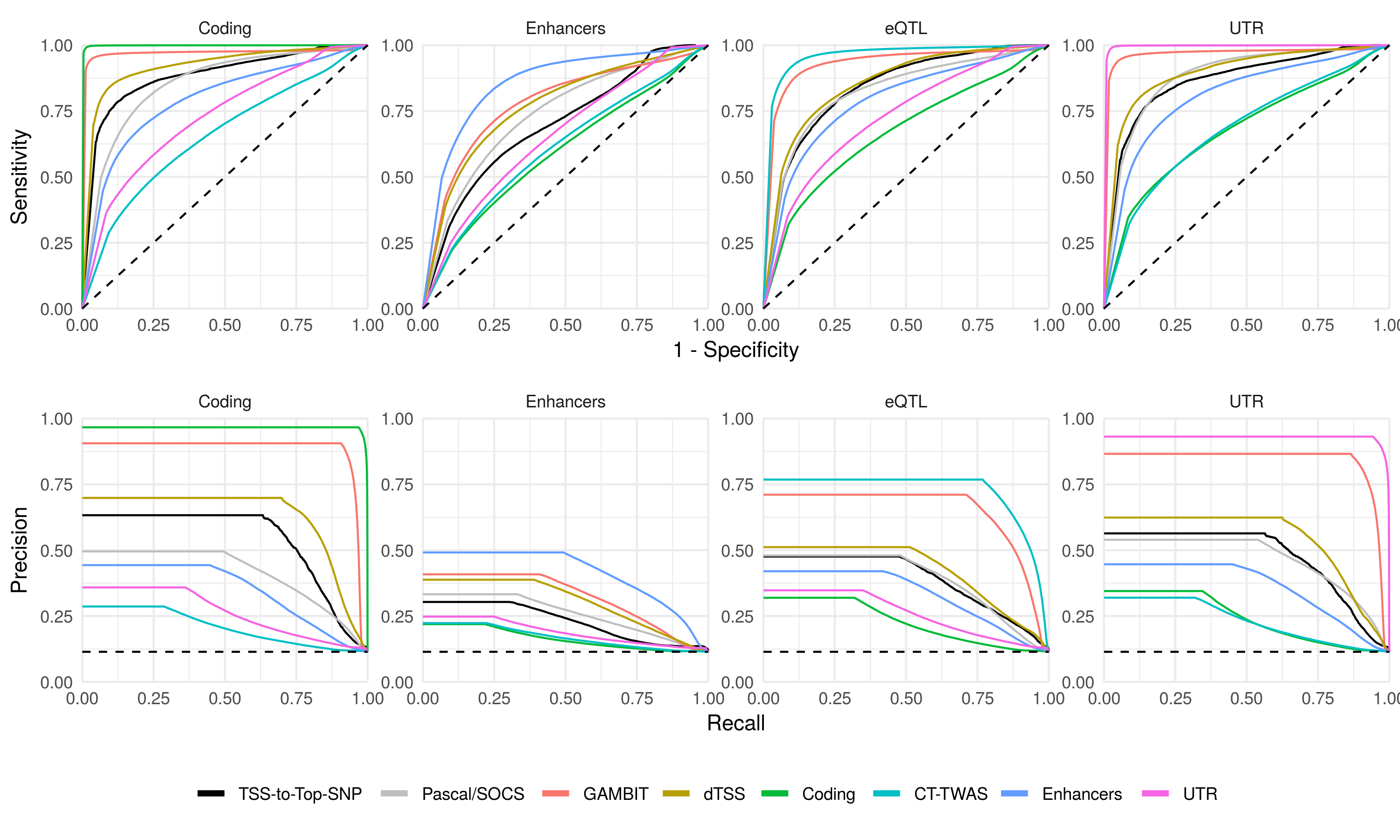

Supplement: S1 Fig — Receiver Operating Characteristic (ROC; top) and Precision-Recall (bottom) curves for each gene-based testing approach (curve color) when either coding, eQTL, enhancer, or UTR variants are causal (plot columns) given locus heritability hL2 = 0.05%; similar results were obtained for other hL2 values. Detailed description of simulation settings is provided under “GWAS Simulations”, and simulation procedures are described in Materials and Methods. To aggregate results across loci and simulation replicates, we use standardized scores for each method calculated by dividing gene-based scores (e.g., -log10-p-values) by the maximum value at the corresponding locus within each replicate. This procedure ensures that curves reflect performance ranking genes at each locus individually. We obtained similar results using the quantile rank of gene-based scores within each locus for each method rather than dividing by the maximum value. (TIF) [file pgen.1009060.s003.tif]

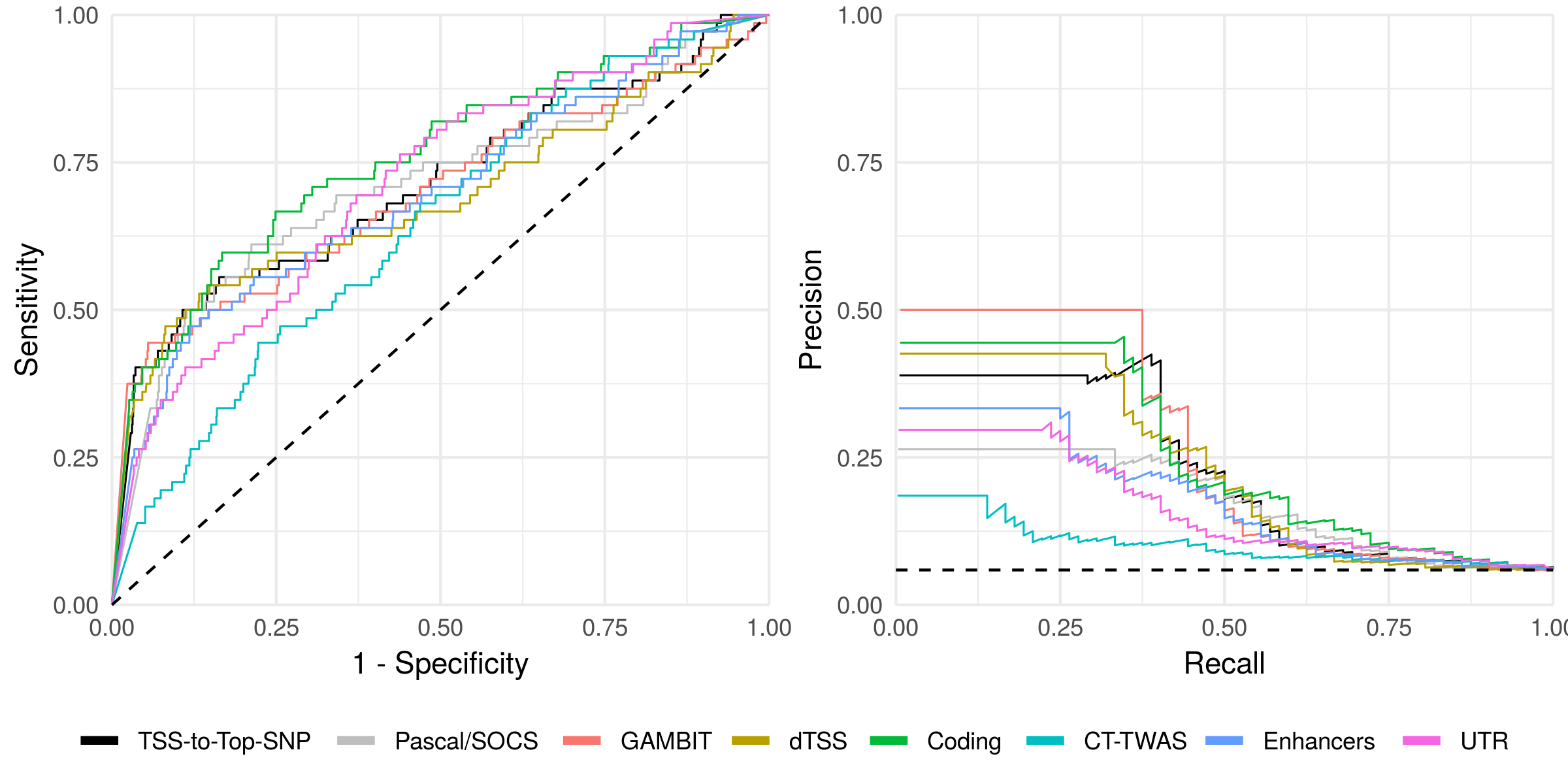

Supplement: S2 Fig — ROC and Precision-Recall curves for each gene-based association or ranking method across benchmark loci present in both HPO and ClinVar (54 loci in total). To aggregate results across benchmark loci and UK Biobank traits, we use standardized scores for each method calculated by dividing gene-based scores (e.g., -log10-p-values) by the maximum value at the corresponding locus. This procedure ensures that curves reflect performance ranking genes at each locus individually. We obtained similar results using the quantile rank of gene-based scores within each locus for each method rather than dividing by the maximum value. (TIF) [file pgen.1009060.s004.tif]

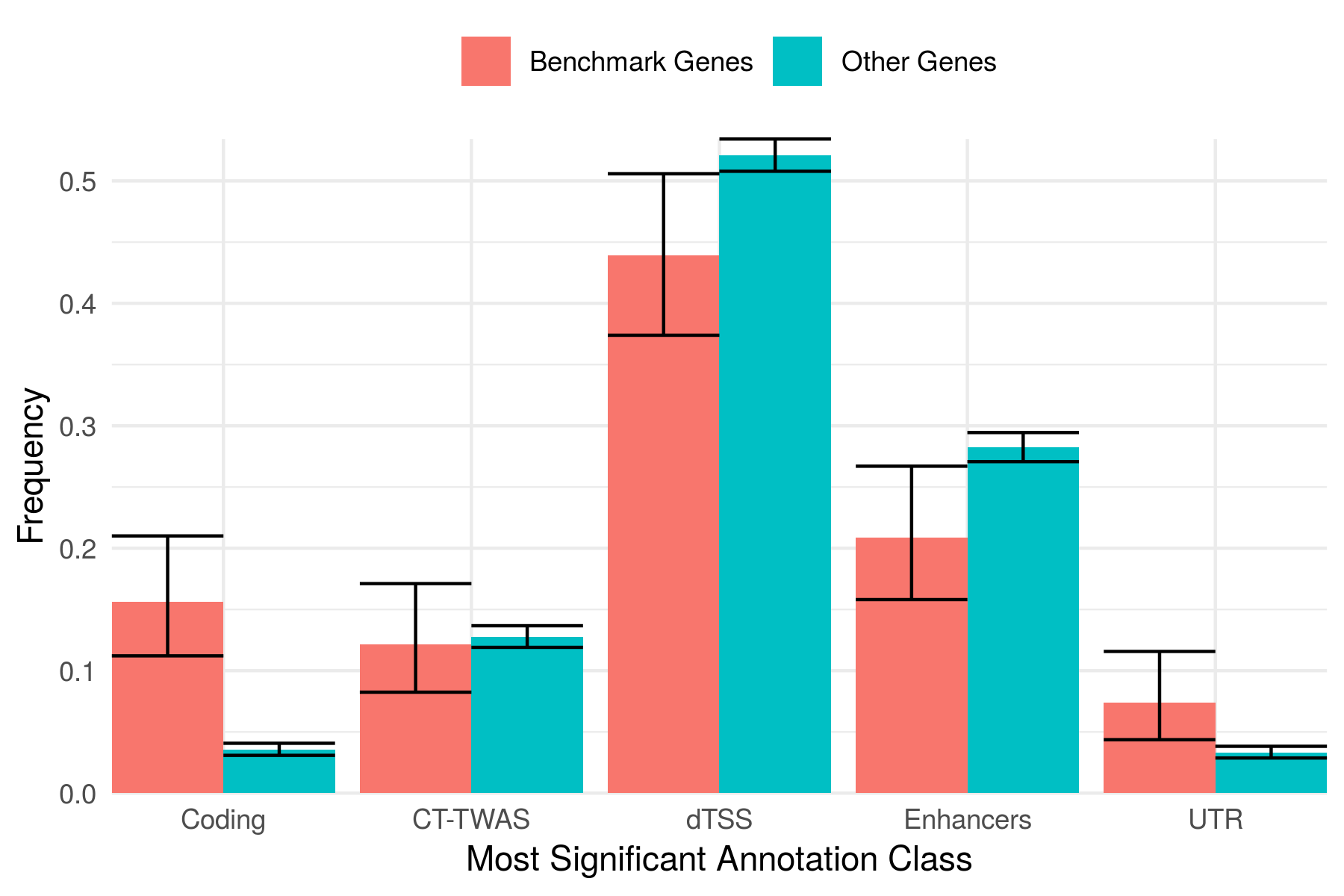

Supplement: S3 Fig — Most significant single-annotation test (x-axis) for genes with one or more gene-based p-value ≤ 5e-6. The proportion of benchmark genes (the union of HPO and ClinVar gene lists) and other genes (not present in either benchmark genes list) for which the indicated annotation class is most significant is shown on the y-axis with 95% confidence intervals. Benchmark genes are strongly enriched for coding associations (odds ratio = 5.03, p-value = 1.3e-16), which is expected due to the selection criteria used to construct benchmark gene lists (described in Materials and methods). (TIF) [file pgen.1009060.s005.tif]

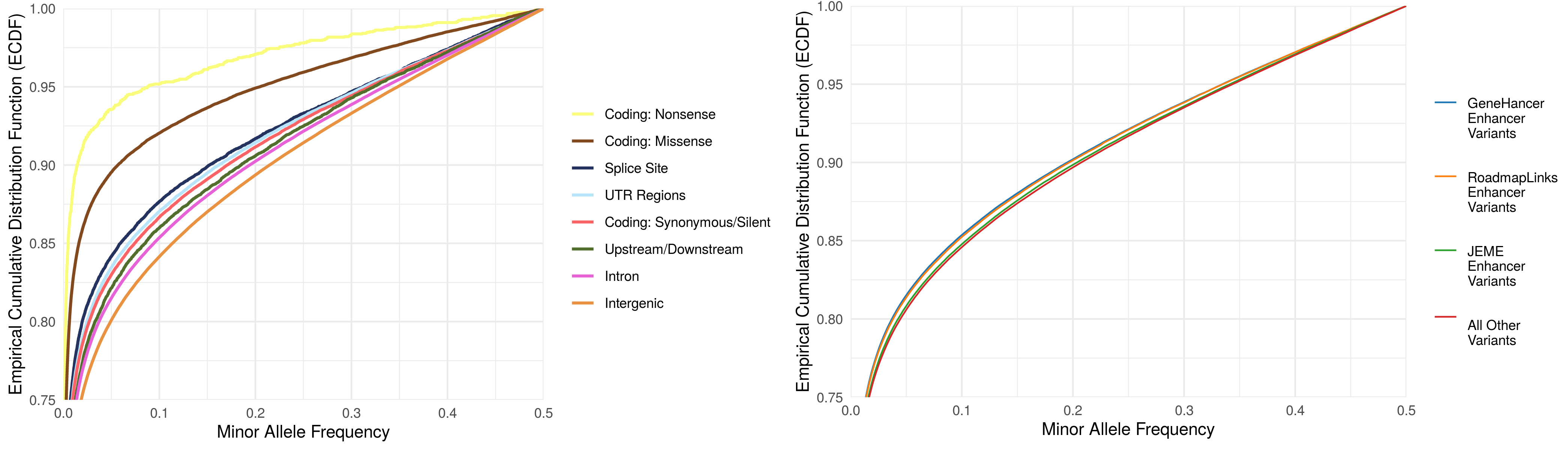

Supplement: S4 Fig — Empirical cumulative distribution function (ECDF) of minor allele frequency (MAF) in the UK Biobank stratified by stratified by functional annotation. Overall, annotated functional variants tend to have lower MAF than intergenic variants, particularly for nonsense and missense variants, as expected. (TIF) [file pgen.1009060.s006.tif]

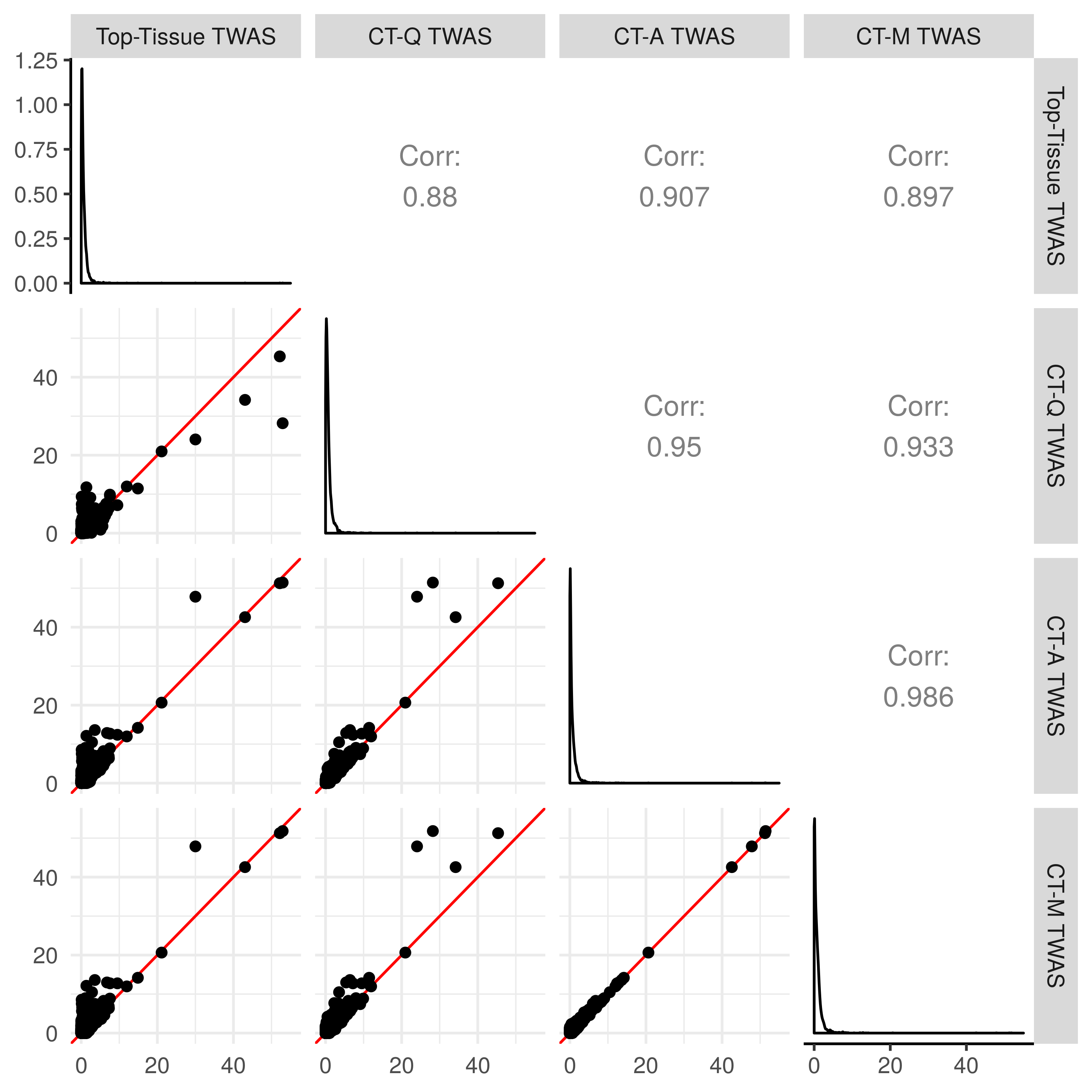

Supplement: S5 Fig — Comparison of Cross-Tissue TWAS (CT-TWAS) p-values, and p-values using only the top single tissue, for disorders of lipoid metabolism using GWAS summary statistics from the UK Biobank. The top tissue was defined as the tissue with the largest number of significant genes using FWER threshold α = 0.05 with Bonferroni adjustment for the number of eGenes in each tissue. In this case, the top tissue was “Liver” with 27 significant genes out of 3,314 total eGenes (Bonferroni-adjusted p-value threshold = 1.5 × 10^−5^). Top-Tissue p-values are compared with CT-TWAS p-values (CT-Q, CT-A, and CT-M), which aggregate across all 47 tissues, restricted to Liver eGenes. CT-Q is calculated using the sum of squared single-tissue TWAS z-scores (similar to SKAT); CT-A is calculated by combining single-tissue TWAS p-values using ACAT; and CT-M is calculated from the minimum single-tissue p-value using the multivariate normal joint density of all single-tissue z-scores (described in Materials and methods). Here, CT-M detected 51 significant genes, followed by CT-A with 47, CT-Q with 33, and top-tissue-only with 27. (TIF) [file pgen.1009060.s007.tif]

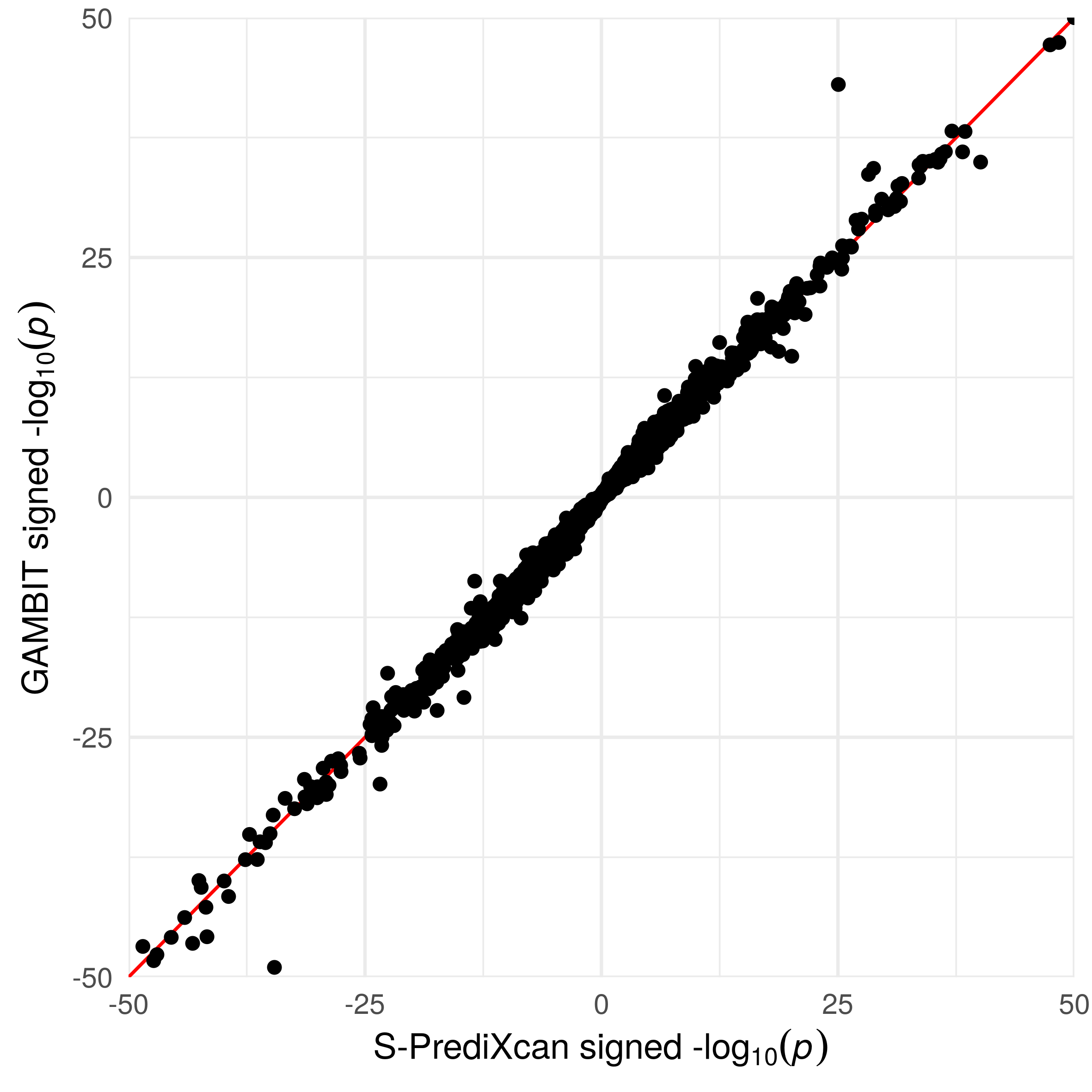

Supplement: S6 Fig — Comparison of TWAS/PrediXcan p-values calculated by GAMBIT versus S-PrediXcan (cloned from GitHub on April 10, 2020) using GWAS summary statistics for HDL cholesterol from the Global Lipids Genetics Consortium [71]. Results are shown for 25,691 unique genes across 47 tissues using GTEx v7 HapMap predictive weights from PredictDB [6, 7]. Signed -log10(p)-values are shown for p ≥ 10−50; 10 genes with outlying p < 10−50 are not displayed. The squared Pearson correlation between z-scores is 0.995; differences in z-scores between GAMBIT and S-PrediXcan are presumably due to differences in the LD reference data. S-PrediXcan uses precomputed LD files which are packaged together with predictive weights, whereas GAMBIT calculates LD interactively from a reference panel (here, European individuals in the 1000 Genomes Project). (TIF) [file pgen.1009060.s008.tif]
